# Ursolic acid drug-drug nanocrystals ameliorate cholestatic liver injury via inhibiting oxidative stress and regulating bile acid metabolism

**DOI:** 10.3389/fphar.2025.1586141

**Published:** 2025-05-30

**Authors:** Manhang Hu, Xiaolu Hua, Wei Xiong, Enhui Zheng, Xingyue Yang, Yu Lu, Bing He, Xiaolin Zhong, Zongzhe Jiang, Qingbi Zhang, Yan Liu

**Affiliations:** ^1^ Basic Medicine Research Innovation Center for Cardiometabolic Diseases, Ministry of Education, Luzhou Municipal Key Laboratory of Thrombosis and Vascular Biology, Metabolic Vascular Diseases Key Laboratory of Sichuan Province, School of Pharmacy, School of Public Health, Southwest Medical University, Luzhou, China; ^2^ Environmental Health Effects and Risk Assessment Key Laboratory of Luzhou, Southwest Medical University, Luzhou, China; ^3^ Department of Science and Technology, Southwest Medical University, Luzhou, China; ^4^ The Public Platform of Advanced Detecting Instruments, Public Center of Experimental Technology, Southwest Medical University, Luzhou, China; ^5^ Department of Gastroenterology, The Affiliated Hospital of Southwest Medical University, Luzhou, China

**Keywords:** ursolic acid, nanocrystals, cholestatic liver injury, VES, oxidative stress, PXR/CYP3A4/UGT1A1

## Abstract

**Introduction:**

Cholestatic liver injury (CLI) is a liver dysfunction closely associated with oxidative stress and bile acid (BA) metabolic disorders, but effective therapies are lacking.The use of ursolic acid (UA) and *α*-Tocopherol succinate (VES) together for treating CLI is promising due to their respective effects on regulating bile acid metabolism and providing antioxidant activity.

**Methods:**

In this study, we synthesized drug-drug nanocrystals (UA-NSps) composed of UA and VES to treat CLI and evaluated their synergistic therapeutic effects by regulating bile acid metabolism and inhibiting oxidative stress in ANIT-induced CLI mice.

**Results and discussion:**

Our investigation demonstrated that UA-NSps exhibited high drug-loading capacity, spherical morphology, and improved dissolution and oral bioavailability. In the ANIT model, UA-NSps effectively restored liver function, as evidenced by histopathological and biochemical improvements. Mechanistically, UA-NSps enhanced Nrf2 nuclear translocation, upregulated Nrf2 and HO-1, reduced pro-inflammatory cytokines, and ameliorated mitochondrial damage. Moreover, UA-NSps alleviated the bile acid metabolism disorders by upregulating the transcriptional activity of UGT2B1, BSEP, and MRP2, as well as the protein expression of nuclear receptors and metabolic enzymes PXR,CYP3A4, and UGT1A1. Our study presents a novel drug-drug nanocrystal strategy that enhances the therapeutic efficacy against CLI by inhibiting oxidative stress and regulating bile acid metabolism.

## 1 Introduction

Cholestasis is a common clinical condition in liver disorders, characterized by a disruption in the production or release of bile from the liver (Gasmi and Kleiner, 2020).Without timely treatment, this condition may progress to liver fibrosis, cirrhosis, and, in severe cases, liver cancer (Smets et al., 2021). Ursodeoxycholic acid (UDCA) and obeticholic acid (OCA) are the primary medicines used to treat primary biliary cirrhosis (PBC) (Harms et al., 2019). However, approximately 33% of patients do not respond to UDCA, and many patients treated with OCA experience severe side effects, such as pruritus and disrupted lipid metabolism (Smets et al., 2021; Harms et al., 2019). Currently, these medications are primarily used for managing PBC, as there are no effective treatments available for other cholestatic disorders (Agrawal et al., 2019; Cabrera et al., 2019). Therefore, it is urgent to develop safe and effective therapeutic agents and strategies.

Disorders in bile acid metabolism and transport are widely regarded as the direct causes of cholestatic liver injury (CLI) (Chiang and Ferrell, 2018). The pregnane X receptor (PXR), a key sensor of harmful substances, plays a critical role in maintaining bile acid homeostasis and has been the focus of extensive research (Fiorucci et al., 2021; Lan et al., 2023). PXR regulates the expression of various detoxification enzymes and transporters, such as UGT1A1 and CYP3A4, through transcriptional control (Lan et al., 2023; Chen et al., 2014). UGT1A1, a predominant conjugating enzyme in phase II metabolism, is crucial for maintaining bile acid, bilirubin, and steroid homeostasis, as well as clearing exogenous metabolites (Kasteel et al., 2020). CYP3A4, a key metabolic enzyme, converts hydrophobic bile acids into hydrophilic forms (Chen et al., 2014). In addition, PXR is mainly expressed in hepatocytes in humans and is expressed at extremely low levels in other cells (Lan et al., 2023).Therefore, the PXR/CYP3A4/UGT1A1 axis is considered a promising therapeutic target for CLI.

Harmful bile acids (BAs) impair mitochondrial activity in liver cells, leading to the excessive production of reactive oxygen species (ROS) (Shu et al., 2019; Yang et al., 2019). Elevated ROS continuously damage the liver, resulting in hepatocyte mitochondrial DNA (mtDNA) damage (Zhang et al., 2019). Thus, the accumulation of harmful BAs is closely associated with oxidative stress-mediated hepatic damage. Nuclear factor erythroid 2-related factor 2 (Nrf2), a key transcription factor, regulates the activation of genes that protect cells from oxidative stress (Jung et al., 2019). Activation of Nrf2 enhances the expression of various antioxidant proteins and peptides, such as heme oxygenase-1 (HO-1) and NAD(P)H oxidoreductase-1 (NQO-1) (Robledinos-Antón et al., 2019). Moreover, Nrf2 is involved in the regulation of genes associated with bile acid metabolism and transport, including UGTs and MRP2 (Zhang et al., 2020). Thus, Nrf2 is also recognized as a critical target in the treatment of CLI.Therefore, targeting oxidative stress and regulating bile acid metabolism pathways could be a promising and potential approach for the treatment of CLI.

Recent research increasingly highlights the multitargeted anticholestatic effects of herbs and natural compounds (Huang et al., 2022).Among these, ursolic acid (UA), a pentacyclic triterpenoid, has gained significant attention for its hepatoprotective properties. Previous studies have demonstrated that UA possesses notable anti-inflammatory effects and can mitigate liver damage by suppressing the TLR4/NF-κB pathway (Ma et al., 2023). Additionally, UA has been shown to regulate bile acid metabolism via the Nrf2/UGT2B7/MRP2/BSEP pathway, offering a potential treatment for CLI (Wang et al., 2024). However, its poor solubility, low permeability and low oral bioavailability limit its clinical application (Jiang et al., 2017; Manayi et al., 2018). To overcome these challenges, researchers have explored various nanocarrier systems for UA, such as liposomes (Wang et al., 2017), polymeric nanoparticles (Antônio et al., 2017), nanocrystals (Pi et al., 2016). However, almost all the above nanopreparations have been developed for cancer treatment (Manayi et al., 2018; Wang et al., 2017), and there have been no reports of UA nanomedicines for the treatment of CLI.

Nanocrystals (NC) are carrier-free colloidal drug delivery systems that contain drug nanoparticles and minimal stabilizers. Due to their small particle size and large specific surface area, NC can increase the solubility and dissolution velocity of poorly soluble drugs. NC is typically produced in the forms of nanosuspensions in an outer liquid phase, usually water, in which they are modified by stabilizers (Jacob et al., 2020). In previous studies, synthetic stabilizers and surfactants such as polyvinyl pyrrolidone (PVP) K90 and sodium dodecyl sulfate (SDS) were often used to prepare UA nanosuspensions (UA-NSps) (Wang et al., 2015). However, problems such as the safety of synthetic stabilizers and surfactants, no therapeutic effect, and increasing the dose of oral drugs in patients have gradually attracted attention (Gajera et al., 2019).Recently, natural stabilizers, such as polysaccharides (Suo et al., 2021), saponins (Chen et al., 2022), and Vitamin E (Wang et al., 2023; Liu et al., 2012; Kesisoglou et al., 2007) have been applied in the preparation of nanosuspensions. These stabilizers can not only address safety problems but also provide additional therapeutic benefits.

Vitamin E, particularly in the form of α-tocopherol succinate (VES), has been identified as a potential supplementary treatment for liver injury due to its antioxidant and anti-inflammatory properties (Kim et al., 2016; Lan et al., 2021). VES has been shown to stabilize nanoparticles and enhance the dissolution rate and oral bioavailability of hydrophobic drugs, such as sorafenib (Wang et al., 2023), carvedilol (Liu et al., 2012), and docetaxel (Kesisoglou et al., 2007). Moreover, VES has demonstrated the ability to alleviate oxidative stress and fibrotic liver damage (Pietu et al., 2012), making it a promising stabilizer for UA nanosuspensions. Therefore, by combining UA with VES, we hypothesize that a drug-drug nanosuspension (UA-NSps) could not only enhance the bioavailability of UA but also offer synergistic effects by targeting both oxidative stress and bile acid metabolism in CLI.

This study aims to develop a novel UA-VES drug-drug nanosuspension and further convert it into solid UA nanocrystals by freeze-drying, then investigate its protective effect on ANIT-induced cholestatic liver injury in mice and reveal the underlying mechanism.

## 2 Materials and methods

### 2.1 Materials

Ursolic acid (purity ≥98%) was purchased from Purifa Technology Co., Ltd. (Chengdu, China). α-Naphthyl isothiocyanate (ANIT), D-α-Tocopherol succinate (VES) and D-α-Tocopherol polyethylene glycol succinate (TPGS) were gained from McLean Biochemical Technology Co., Ltd. (Shanghai, China). The enzyme-linked immunosorbent assay (ELISA) kits of TNF-α and IL-6 were purchased from MEIMIAN Technology (Wuhan, China). HO-1, UGT1A1, CYP3A4, and PXR antibodies were obtained from Sanying Biotechnology Co., Ltd. (Wuhan, China), Nrf2 antibodies were gained from Cell Signaling Technology (Massachusetts, United States), The enzyme-linked immunosorbent assay (ELISA) kits of SOD, MDA, CAT, and GSH-Px were purchased from Nanjing Jiancheng Bioengineering Institute (Nanjing, China).

### 2.2 Preparation and process optimization of nanosuspensions

UA-NSps were prepared using the antisolvent precipitation technique. Briefly, specific amounts of UA and VES were dissolved in the organic phase, while TPGS was dissolved in the aqueous phase. Then, 1 mL of the organic phase was slowly added to the aqueous phase under vigorous stirring at 1,000 rpm for 5 min, followed by ultrasonic treatment for 15 min at 25°C. The organic solvents were subsequently evaporated using a dry nitrogen-blowing concentrator. Finally, the freshly prepared nanosuspensions were lyophilized using a freeze dryer (LABCONCO, United States). In this study, single-factor analysis was employed to optimize the preparation process, with particle size and PDI used as the primary evaluation indices. The effects of different organic solvents, stabilizer dosages, and stabilizer-to-drug ratios on the preparation process were systematically investigated.

### 2.3 Physicochemical characterization of nanosuspensions

Particle size, polydispersity index (PDI), and zeta potential were measured using the dynamic light scattering (DLS) method on a Zetasizer Nano ZS (Malvern Instruments, Malvern, United Kingdom). The stability of UA-NSps stored at 25°C for 3 weeks was assessed by monitoring changes in particle size and PDI.

The morphology of UA-NSps was observed using a transmission electron microscope (TEM) (FEI TECNAI G2 12, United States). Samples were diluted with ultrapure water, placed on a copper grid, dried at room temperature, and negatively stained with 2% phosphotungstic acid (w/v) before observation.

The crystalline state of UA in different samples was analyzed using a D8 ADVANCE X-ray diffractometer (Bruker, Germany) with CuK*α* radiation. Data were collected over a range of 3°–40° at a scanning speed of 2°/min (Singh et al., 2019).

A Fourier transform infrared (FTIR) spectrophotometer (Shimadzu, model IR Affinity-1S) was used to analyze the interactions among components. Scans were performed across wavelengths ranging from 4,000 cm^−1^ to 400 cm^−1^.

Approximately 5 mg of each sample was accurately weighed and placed in aluminum pans for differential scanning calorimetry (DSC) analysis. DSC thermograms were recorded at a heating rate of 10 °C/min over a temperature range of 100°C–350°C under a nitrogen atmosphere.

### 2.4 Optimization of lyophilization for nanosuspensions

Mannitol, galactose, and glucose at a concentration of 0.5% (w/v) were each added to the UA-NSps solution, frozen at −80°C, and subsequently lyophilized for 48 h. Glucose at concentrations of 0.5%, 1%, 2%, and 5% (w/v) was separately added to UA-NSps and lyophilized using the same procedure. The lyophilized powders were reconstituted with 1 mL of deionized water, and the particle size and PDI were determined using DLS (He et al., 2023).

### 2.5 Drug loading capacity (DLC)

DLC was determined using high performance liquid chro-matography (HPLC).

UA-NSps lyophilized powder was dissolved in methanol and demulsified by ultrasound for 5 min. The above solution was filtered by 0.45 μm filter membrane, and the free drug contents were determined by HPLC. The DLC was obtained via the following formula:

DLC%=W1/W2×100%

Where W1 and W2 are the weight of drug in UA-NSps and the weight of the UA-NSps, respectively.

### 2.6 *In vitro* release of UA-NSps

The dissolution of the samples was evaluated using the Chinese Pharmacopeia Type II method with a dissolution tester (RC-80F, Tianjin Chuangxing Electronic Equipment Manufacturing Co., Ltd.). The dissolution medium consisted of PBS (pH 6.8) with 0.2% SDS (w/v). Pure UA powder and lyophilized UA-NSps powder (equivalent to 50 mg of UA) were added to 450 mL of the dissolution medium and stirred at 37°C using 200 rpm. At predetermined intervals, 1 mL of the sample was withdrawn, filtered by 0.45 µm filter membrane, and analyzed for UA concentration using HPLC.

### 2.7 *In vivo* pharmacokinetic study

A total of 60 C57BL/6J mice were randomly assigned into two groups, namely, the oral pure UA group and the UA nanosuspension group, with each group consisting of 30 mice. Prior to the experiment, mice were fasted for 24 h, and the dosage of UA administered was 40 mg·kg^−1^, which was calculated based on the drug loading. Blood samples from the orbital sinus and liver tissue were collected at predetermined time points of 0, 0.5, 1, 1.5, 2, 4, 5, 6 and 12 h after administration and transferred to centrifuge tubes. The collected blood samples were centrifuged at 5000 rpm for 10 min to separate the supernatant and obtain plasma. Appropriate amount of liver tissue was homogenized with twice its volume of normal saline. Subsequently, 100 μL of biological samples was fully mixed with 300 μL of acetonitrile and centrifuged at 10,000 rpm for 10 min. The supernatant was filtered through a 0.45 μm aqueous phase filter membrane and subsequently analyzed using HPLC. Pharmacokinetic parameters were calculated using DAS 2.0 software.

### 2.8 Animals and experimental design

Male C57BL/6J mice (6–8 weeks of age, 20 ± 2 g) were obtained from Beijing Hua Fukang Biotechnology Co., Ltd. (SCXK 2018-0008, China). All animal experimental procedures were approved by the Ethics Committee of Southwest Medical University (Project Identification Code: 20230112-001). The mice were housed in a standard animal facility under controlled conditions (temperature: 20°C–25°C) with a 12-h light/dark cycle. The mice were randomly divided into five groups (n = 7 per group): a control group, an ANIT group, and three treatment groups (UA, UA-NSps, and physical mixture). For the treatment groups, intragastric administration was performed once daily for 14 days at a dose of 40 mg/kg for UA, UA-NSps, and the physical mixture. The control group received an equal volume of normal saline. To induce cholestasis, on the 12th day of treatment, the ANIT group and the three treatment groups were given an intragastric dose of ANIT dissolved in olive oil (50 mg/kg), while the control group received an equal volume of olive oil (Zhang et al., 2020). Body weight was measured daily throughout the experiment.

### 2.9 Hepatic histopathological examination

Liver tissues were fixed in 4% paraformaldehyde (PFA), embedded in paraffin, and sliced into 5 μm sections for HE staining.

### 2.10 Serum biochemical assay

The blood samples were centrifuged at 3,000 rpm for 15 min to obtain serum. Automated standardized procedures were used to measure the serum levels of aspartate aminotransferase (AST), alanine aminotransferase (ALT), alkaline phosphatase (ALP), total bile acid (TBA), total bilirubin (T-BIL), and γ-glutamyl transpeptidase (γ-GT).

### 2.11 Antioxidase assay

The contents of superoxide dismutase (SOD), malondialdehyde (MDA), glutathione peroxidase (GSH-Px), and catalase (CAT)were detected by ELISA in liver tissues.

### 2.12 Electron microscopy

Liver tissues were fixed with 3% glutaraldehyde and 1% osmium tetroxide, dehydrated with acetone solutions, infiltrated with Epox 812, embedded and cut into ultrathin sections, which were then examined with a transmission electron microscope.

### 2.13 RT-qPCR

Total RNA was extracted from frozen liver tissues using Trizol Reagent (TransGen Biotech, beijing, China) following the manufacturer’s instructions. The concentration and purity of RNA were determined with a Microplate Reader, with samples having an A260/A280 ratio between 1.8 and 2.0 considered to be of acceptable quality and integrity.Subsequently, the RNA was reverse-transcribed into cDNA using the reverse transcription system kit (Takara, Osaka, Japan). RT-qPCR analysis was performed using TB Green Master Mix (Takara, Osaka, Japan) on an Applied Biosystems Quant Studio system (Thermo Fisher, Germering, Germany). PCR amplification results were analyzed using DATA assist™ software. GAPDH was used as the internal reference, and the mRNA expression levels were calculated using the 2^−ΔΔCT^ formula. The primers sequences corresponding to the genes investigated in the present study are listed in Supplementary Table S1.

### 2.14 Immunofluorescence

Immunofluorescence was performed on maximal surface sections of paraffin-embedded liver tissue from C57 mice. Sections were deparaffinized using Deparaffinization Solutions I–III (Servicebio, China) for 10 min each, rehydrated through a graded ethanol series (anhydrous ethanol I–III, 5 min each), and rinsed with distilled water. Antigen retrieval was carried out in EDTA buffer (pH 8.0) using a microwave oven for 15 min.After cooling to room temperature, tissue areas were circled with a hydrophobic barrier pen and blocked with 5% BSA for 30 min. Sections were incubated overnight at 4°C with rabbit anti-Nrf2 primary antibody (1:1000, GB113808, Servicebio, China). Following three washes with PBS (pH 7.4), Cy3-conjugated goat anti-rabbit IgG secondary antibody (1:300, GB21303, Servicebio, China) was applied for 50 min at room temperature in the dark.Nuclei were counterstained with DAPI, and sections were washed again with PBS. Autofluorescence was quenched using Solution B (Servicebio, China) for 5 min, followed by rinsing under running water for 10 min. Slides were mounted with antifade medium and imaged using a fluorescence microscope (ECLIPSE C1, Nikon, Tokyo, Japan).

### 2.15 Western blotting

Frozen liver tissues were homogenized in RIPA lysis buffer containing protease inhibitor (Beyotime, China), then separated via 7.5%–10% SDS‒PAGE, followed by transferring to PVDF membranes. The transferred membranes were incubated with specific primary antibodies (HO-1 (1:1000), Nrf2 (1:1000), UGT1A1 (1:1000), PXR (1:1000), CYP3A4 (1:1000)) and the secondary antibody (1:10,000). Finally, the incubated antibody bands were observed in a chemiluminescence imaging device (ChemiDoc XRS+, Bio-Rad, United States).

### 2.16 Statistical analysis

GraphPad Prism 8.0 (version 8.0, La Jolla, CA, United States) was used for all the statistical analyses. The results were expressed as the mean ± standard error (Mean ± SEM) of independent three experiments.T tests and one-way ANOVA were applied to analyze statistical differences among groups A *p*-value <0.05 was considered statistically significant.

## 3 Results and discussion

### 3.1 Preparation and process optimization of UA-NSps

According to Supplementary Table S1, methanol was selected as the organic phase. The optimal mass ratio of UA to VES was determined to be 5:3, with a final UA concentration of 0.3 mg/mL. Additionally, the amount of TPGS used was set at 1 mg.When VES was used alone to prepare UA-NSps (Supplementary Table S1B), some nanoparticles were observed to aggregate under electron microscopy. To address this issue, we employed TPGS as a co-stabilizer, based on prior research (Liu et al., 2012).

### 3.2 Characterization of UA-NSps

**FIGURE 1:**
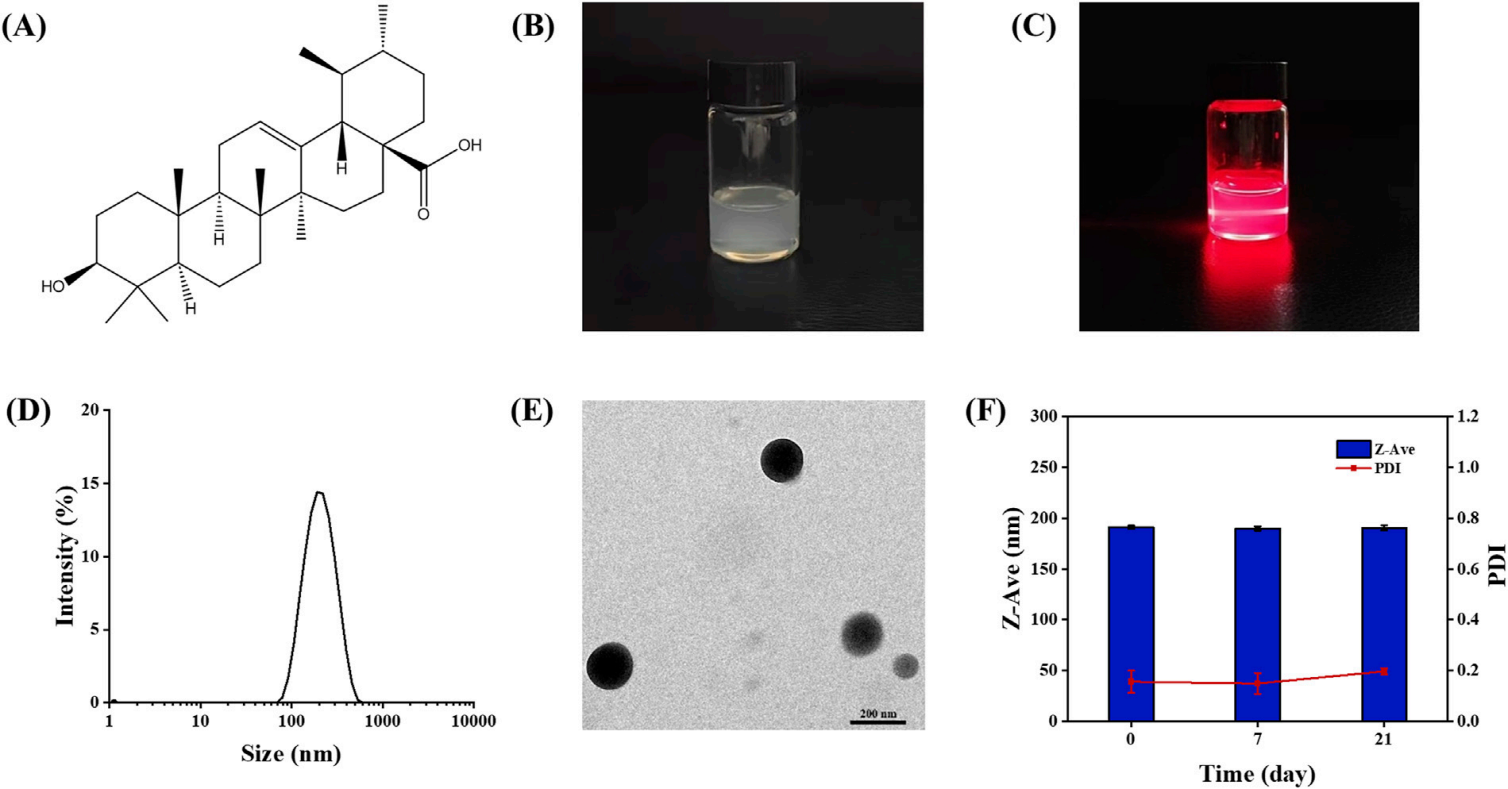
**(A)** Chemical structure of Ursolic acid.**(B)** Images **(C)**Tyndall Effect and **(D)** Particle size distribution of UA-NSps prepared under optimal conditions. **(E)** TEM of UA-NSps. **(F)** Stability of UA-NSps during 3 weeks under 25°C.

The chemical structure of UA is shown in Figure 1A. The freshly prepared UA-NSps appeared as a white liquid exhibiting the Tyndall effect (Figures 1B,C). The particle size distribution curve (Figure 1D) indicated a mean particle size of 187.8 ± 5.9 nm, with a PDI of 0.156 ± 0.044, demonstrating uniform dispersion. The mean zeta potential was -22.7 ± 0.603 mV, indicating excellent physical stability. TEM images (Figure 1E) revealed that the particles were monodisperse and had a uniform spherical shape. In the stability experiments, UA-NSps maintained a stable particle size and uniform dispersion over 3 weeks at 25°C (Figure 1F).

**FIGURE 2:**
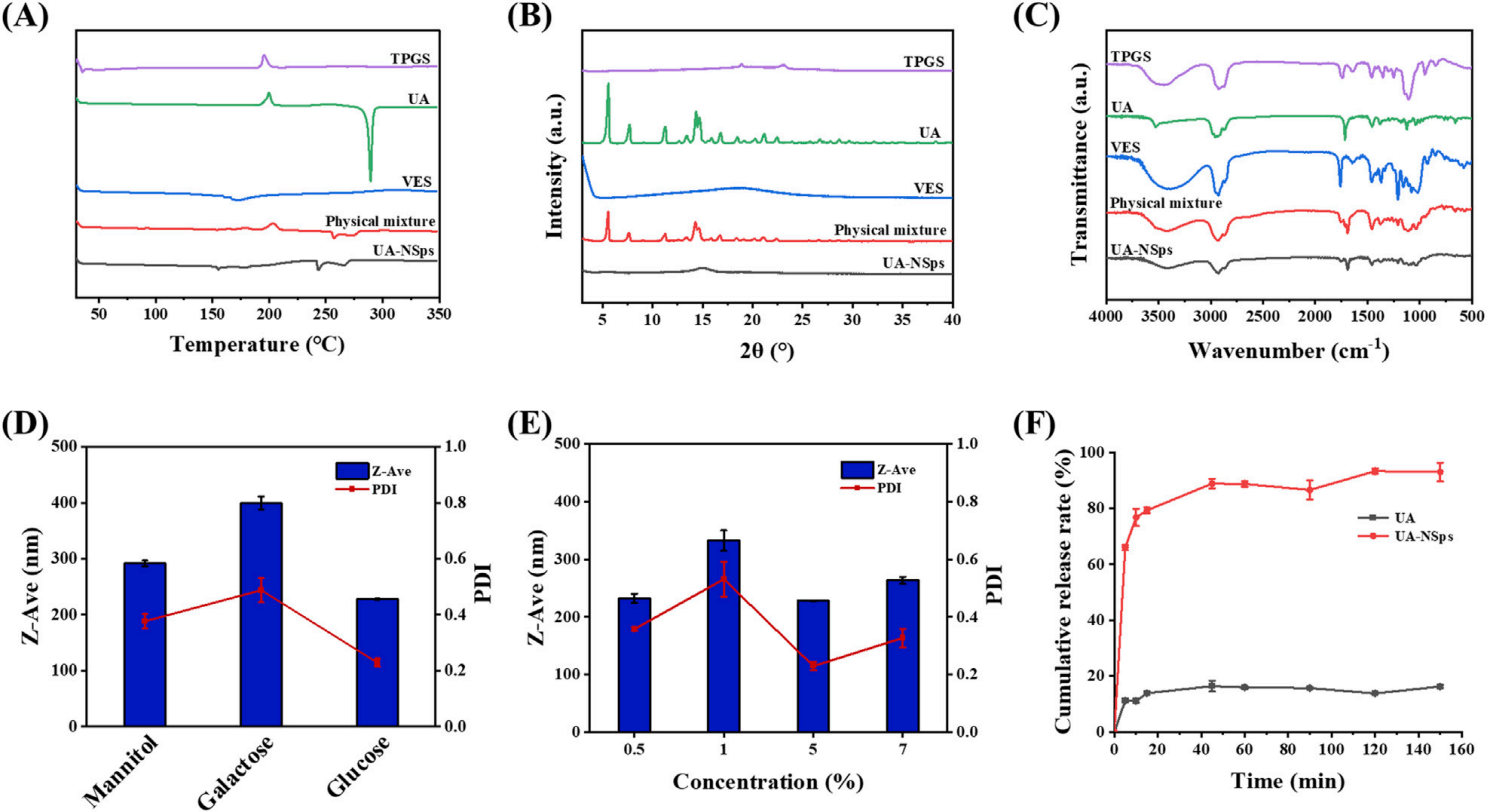
**(A)** Differential scanning calorimetry thermograms. **(B)** X-ray diffractograms and **(C)** FTIR spectra of TPGS, UA, VES, physical mixture and UA-NSps. **(D)** Particle size and PDI of UA-NSps lyophilized powders prepared with different cryoprotectants. **(E)** Particle size and PDI of UA-NSps lyophilized powders prepared with different concentrations of glucose. **(F)** Release profiles of raw UA and UA-NSps in phosphate-buffered saline (pH = 6.8) at 37°C ± 0.5°C (mean ± SEM, n = 3).

DSC and XRD analyses were conducted to examine the crystalline structure of UA in UA-NSps. The DSC thermograms (Figure 2A) showed that pure UA exhibited a distinct endothermic peak at 288.40°C, corresponding to its crystalline structure. VES displayed a broad endothermic peak at approximately 160°C. In contrast, the exothermic peak of UA disappeared in the UA-NSps profile, and the endothermic peak at 288.40°C shifted to 243.32°C with reduced intensity. This indicated that UA particles were tightly encapsulated by the stabilizers.

The XRD profiles (Figure 2B) showed typical crystalline peaks of UA at 5.6°, 7.8°, 11.3°, 14.4°, 14.7°, 16.8°, 21.1°, and 22.4° in pure UA and the physical mixtures, confirming the crystalline nature of UA. However, these peaks were absent in the UA-NSps profile, indicating that UA underwent a transformation from a crystalline to an amorphous state during the preparation process. In conclusion, the DSC and XRD analyses confirmed the crystalline-to-amorphous transformation of UA in UA-NSps (Singh et al., 2019).

FTIR analysis was used to investigate potential intermolecular interactions between UA and the excipients. The FTIR spectrum of UA (Figure 2C) displayed distinct peaks corresponding to O-H stretching (3510 cm^−1^), C-H stretching (2940 cm^−1^), and C=O stretching (1693 cm^−1^). The UA-NSps spectrum showed overlapping peaks of UA, TPGS, and VES, without any significant spectral shifts. This indicated no significant interaction between the drug and the excipients, and the molecular structure of UA remained unchanged during the formulation of the nanosuspensions (Pi et al., 2016).

### 3.3 Lyophilization process of UA-NSps

The freeze-drying method is commonly employed to obtain solid nanocrystals, which is convenient for their storage, transportation, and commercial-scale production (He et al., 2023). Frequently, nanoparticles tend to clump together during freeze-drying,leading to difficulties in redispersion in water. The addition of freeze-drying stabilizers can effectively prevent nanoparticle aggregation and preserve the original drug structure (He et al., 2023). Different types and concentrations of cryoprotectants were used to prepare the UA-NSps lyophilized powders in this study. Figures 2D,E showed that the lyophilized powders containing 5% (w/v) glucose as a cryoprotectant exhibited minimal variations in particle size and PDI before and after re-dispersion, indicating excellent redispersibility. Therefore, 5% (w/v) glucose solution was chosen as the cryoprotectant for UA-NSps.

### 3.4 DLC and drug release analysis

The UA content was determined by HPLC using a standard curve (*y* = 394.35*x*+2.9094, *R* ^*2*^ = 1; Supplementary Figure S1). The DLC calculated from the standard curve was 45.32% ± 2.68%, which was slightly lower than the expected drug loading of 51.72%, possibly due to losses during transfer.

PBS (pH 6.8) containing 0.2% (w/v) SDS was used as the release medium. The drug release curve showed that UA-NSps exhibited rapid initial release within the first 10 minutes, reaching 76.71%, followed by gradual sustained release until reaching a plateau at 88.84% at 45 min (Figure 2F). In contrast, only approximately 20% of UA was released from pure UA within 2 hours, which was significantly lower than that observed for the UA-NSps group. As drug dissolution significantly impacts bioavailability, we conclude that UA-NSps enhanced the dissolution of UA and improved its bioavailability, thereby increasing its therapeutic efficacy.

### 3.5 Pharmacokinetic study

**FIGURE 3:**
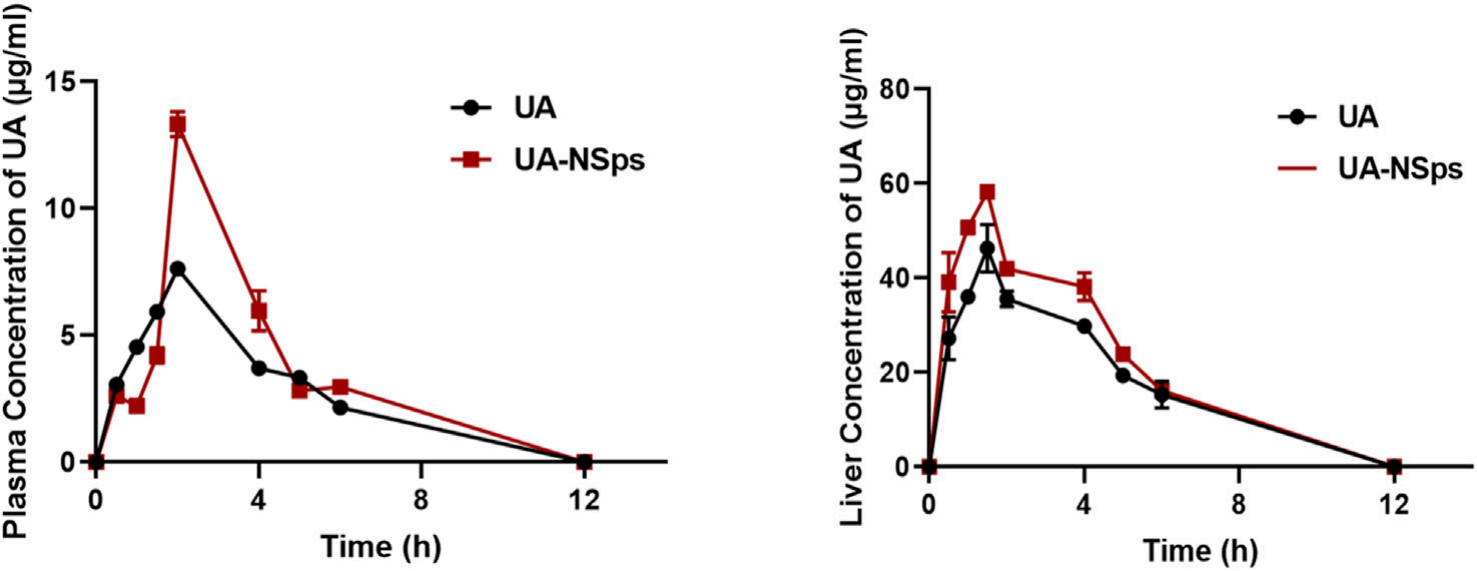
Plasma and liver concentration-time curves of orally administered pure UA and UA nanosuspension (mean ± SEM, n = 3).

Figure 3 revealed that UA-NSps achieved a much higher maximum blood concentration (13.332 ± 0.485 μg/mL) and maximum liver concentration (58.203 ± 0.965 μg/mL) *in vivo* compared to the pure UA group. The critical pharmacokinetic parameters (Tables 1, 2) demonstrated that the plasma and liver AUC_0_–t values of UA-NSps were 1.31-fold and 1.26-fold higher, respectively, than those of the UA group. These results indicate that the bioavailability of UA was significantly enhanced upon formulation into a nanosuspension. Furthermore, the clearance rate (CL) of UA-NSps was lower than that of UA, suggesting that the nanosuspension formulation could prolong the drug action time by reducing the clearance rate of UA in mice.

**TABLE 1:** Pharmacokinetic parameters in C57BL/6J mice plasma for each group (n = 3).

Sample	Free UA	UA-NSps
Dose (mg/kg)	40	40
Cmax (µg/mL)	7.631 ± 0.058	13.332 ± 0.485**
T_1/2_ (h)	2.509 ± 0.338	2.53 ± 0.186
CL_z_ (L/h/kg)	1.173 ± 0.056	1.018 ± 0.063
AUC_0-t_ (mg/L*h)	26.263 ± 0.212	34.44 ± 0.912**
MRT_0-t_ (H)	2.737 ± 0.010	2.816 ± 0.420

Significant differences compared with the free UA, group, **p < 0.01.

**TABLE 2:** Pharmacokinetic parameters in C57BL/6J mice liver for each group (n = 3).

Sample	Free UA	UA-NSps
Dose (mg/kg)	40	40
Cmax (µg/mL)	46.281 ± 5.009	58.203 ± 0.965
T_1/2z_ (h)	2.469 ± 0.426	2.29 ± 0.424
CL_z_ (L/h/kg)	0.178 ± 0.016	0.146 ± 0.005**
AUC_0-t_ (mg/L*h)	171.029 ± 5.183	215.679 ± 4.259**
MRT_0-t_ (H)	2.75 ± 0.043	2.29 ± 0.424

### 3.6 UA-NSps treatment improved liver function in mice with ANIT-induced cholestasis

**FIGURE 4:**
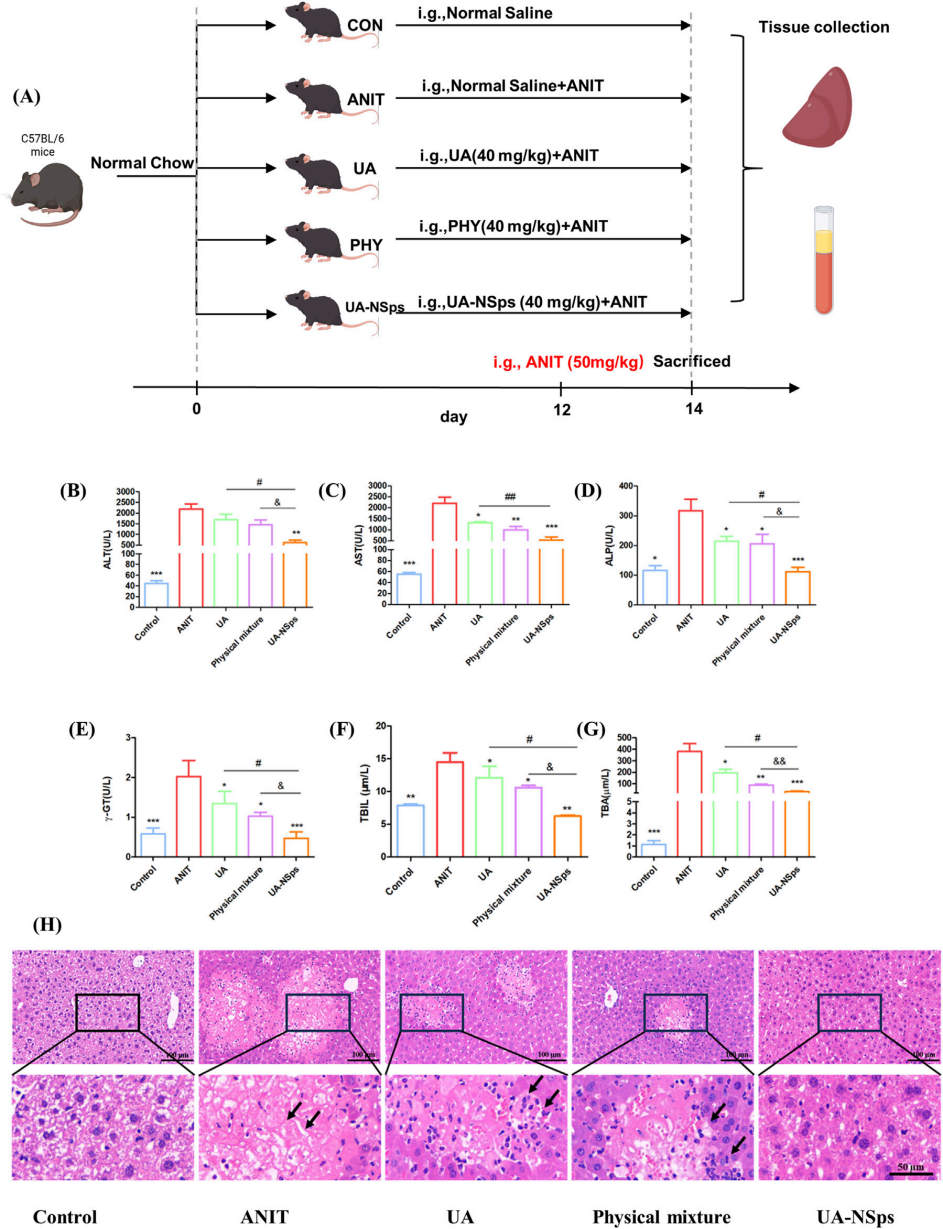
UA-NSps therapy improved liver function in mice with ANIT-induced cholestasis. **(A)** Outline of the experimental scheme. **(B–G)** Serum levels of ALT, AST, ALP, *γ*-GT, TBIL and TBA. Significant differences compared with the ANIT group, *p < 0.05, **p < 0.01, ***p < 0.001; compared with the UA group, ^#^ *p* < 0.05, ^##^ *p* < 0.01; compared with the physical mixture group, ^&^ *p* < 0.05, ^&&^ *p* < 0.01; **(H)** Representative images of H&E-stained liver sections (scale bars:100 µm and 50 µm). Data are presented as the mean ± SEM (n = 7). The arrows indicate hepatocellular necrosis.

The experimental schemes are shown in Figure 4A. To evaluate the hepatoprotective effect of UA-NSps, we measured the levels of ALT, AST, ALP, as well as cholestasis markers including TBIL, TBA, and *γ*-GT. As shown in Figures 4B–G, the ANIT group showed significant increases in ALT, AST, ALP, TBIL, TBA, and γ-GT levels (*p* < 0.05), which were partially reversed after treatment with UA and the physical mixture (*p >* 0.05), and significantly reversed after treatment with UA-NSps (*p* < 0.05). The physical mixture treatment exhibited higher potency than UA in reducing all these specific markers. Notably, the UA-NSps group had better therapeutic efficacy than the UA group and the physical mixture group (*p* < 0.05)

**FIGURE 5:**
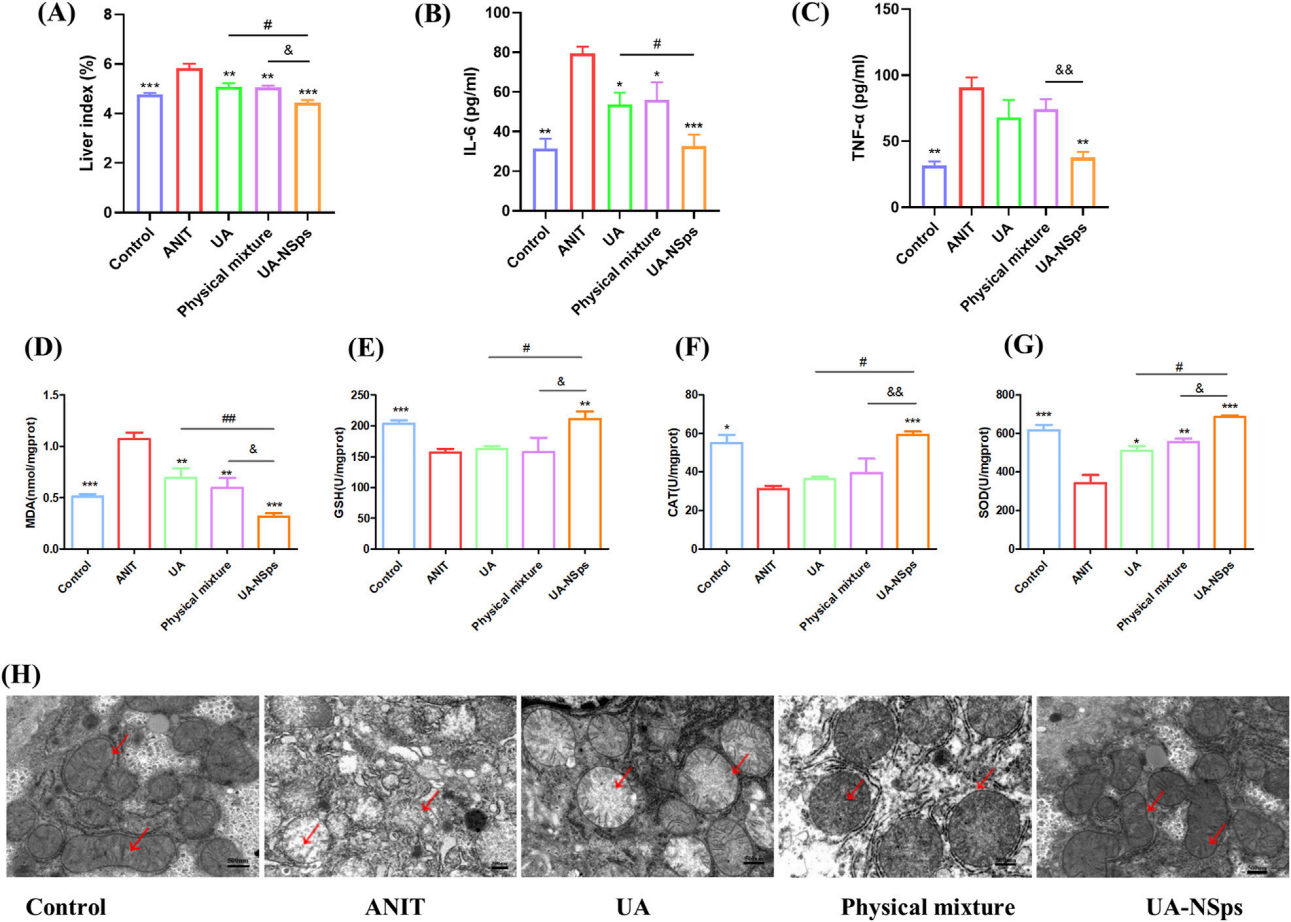
UA-NSps suppressed liver oxidative stress in mice with ANIT-induced cholestasis. **(A)** UA-NSps therapy improved liver index. **(B)** interleukin-6 (IL-6), **(C)** tumor necrosis factor-*α*(TNF-α) levels in serum. **(D–G)** MDA, GSH, CAT and SOD levels in the liver. Data are presented as the mean ± SEM (n = 3). Significant differences compared with the ANIT group, *p < 0.05, **p < 0.01, ***p < 0.001; compared with the UA group, ^#^ *p* < 0.05, ^##^ *p* < 0.01; compared with the physical mixture group, ^&^ *p* < 0.05, ^&&^ *p* < 0.01; **(H)** UA-NSps alleviated ANIT-induced mitochondrial damage. (scale bar: 500 nm). The red arrows indicate mitochondrial membranes and cristae.

Hepatomegaly is a key clinical feature of CLI, and the liver index is commonly used to assess hepatomegaly (Yuan et al., 2022). After a 2-week experimental period, the liver index of mice in the ANIT group was significantly increased compared to the control group (Figure 5A). Compared with UA and physical mixture groups, the liver index of mice in the UA-NSps group was markedly reduced and nearly restored to the normal level (*p* < 0.05).

Furthermore, histopathological evaluation revealed extensive hepatocyte necrosis and inflammation in the liver tissues of the ANIT group (Figure 4H). After treatment, hepatocyte necrosis and inflammation were still observed in the UA and physical mixture groups. In contrast, tissue necrosis and inflammation levels were significantly reduced in the UA-NSps group, showing no obvious damage. These results suggested that UA-NSps could effectively ameliorate ANIT—induced cholestasis.

### 3.7 UA-NSps suppressed liver oxidative stress in mice with ANIT-induced cholestasis

Oxidative stress and inflammation are closely linked, with ROS and oxidative stress byproducts enhancing pro-inflammatory responses (Zhang et al., 2019). Inflammation is closely associated with liver damage in cholestasis, and excessive inflammation can exacerbate disease progression (Zhang et al., 2019). ANIT-induced cholestasis can exacerbate liver damage by upregulating pro-inflammatory cytokines such as IL-6 and TNF-α (Yuan et al., 2022). Following the UA-NSps intervention (Figures 5B,C), a significant downregulation of pro-inflammatory factor expression was observed (*p* < 0.05).

ANIT-induced oxidative damage leads to lipid peroxidation and MDA formation. ANIT exposure resulted in significantly increased liver MDA content (*p* < 0.01), indicative of oxidative stress. Additionally, ANIT inhibited the activities of antioxidant enzymes SOD, GSH, and CAT (Figures 5D–G). However, treatment with UA-NSps counteracted the inhibitory effect of ANIT on antioxidant enzyme activity, concurrently reducing MDA levels (*p* < 0.01) (Figure 5D). The improvement in the above indicators was greater in the physical mixture group than in the UA group, but there was no statistically significant difference. Importantly, UA-NSps demonstrated a significantly stronger effect than either UA or the physical mixture in restoring SOD, GSH, and CAT levels (*p*<0.05).

Inflammation and oxidative stress are known to contribute to mitochondrial dysfunction and oxidative damage to mitochondria (Zhang et al., 2019). Mitochondrial morphology was examined using TEM. As shown in Figure 5H, the control group exhibited an intact double-layered mitochondrial membrane and well-defined mitochondrial cristae. In contrast, the ANIT group displayed swollen mitochondria with damaged or absent cristae. While treatment with UA and the physical mixture partially alleviated these mitochondrial changes, administration of UA-NSps significantly mitigated mitochondrial damage, effectively restoring the mitochondrial cristae structure to near-normal levels, comparable to the control group.These results suggested that UA-NSps may mitigate ANIT-induced liver damage by modulating oxidative stress and inflammatory responses.

### 3.8 UA-NSps alleviate ANIT-induced hepatic oxidative damage by activating the Nrf2 antioxidant pathway

**FIGURE 6:**
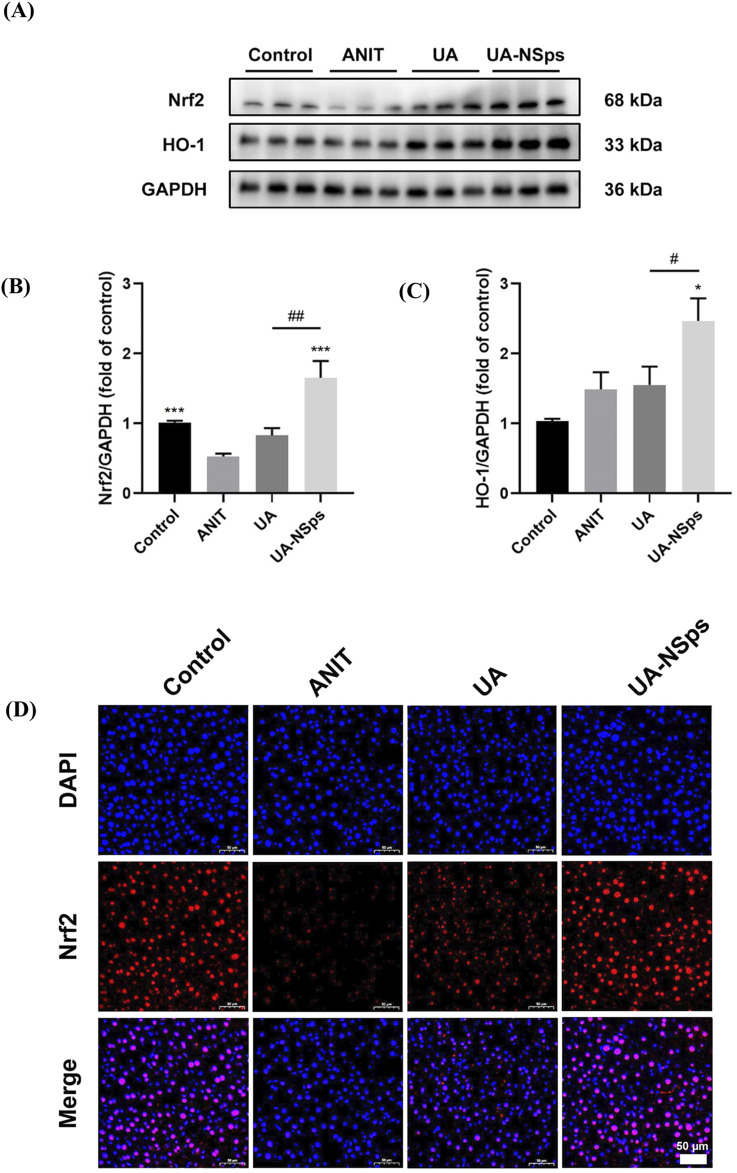
UA-NSps regulated the Nrf2/HO-1 signaling pathway. **(A)** Immunoblot assays of Nrf2 and HO-1. **(B,C)** Relative densities of Nrf2 and HO-1. Data are presented as the mean ± SEM (n = 3). Significant differences compared with the ANIT group, *p < 0.05, **p < 0.01, ***p < 0.001; compared with the UA group, ^#^ *p* < 0.05, ^##^ *p* < 0.01. **(D)** Immunofluorescence staining of Nrf2. (scale bars: 50 *μm*).

The Nrf2/HO-1 signaling pathway plays a critical role in regulating antioxidant defenses (Zhang et al., 2020). To explore the potential mechanism underlying UA-NSps’ protective effects on CLI, the protein levels of Nrf2 and HO-1 were evaluated using Western blotting. Nrf2 expression was significantly reduced in the ANIT group compared to the control group (*p* < 0.05). However, UA-NSps treatment significantly increased the protein levels of Nrf2 and HO-1 (*p* < 0.05) (Figures 6A–C). To further assess Nrf2 activation, nuclear translocation of Nrf2 was examined using immunofluorescence. The results revealed that, compared to the control group, nuclear Nrf2 expression was significantly diminished in the ANIT group. Administration of UA-NSps, however, significantly enhanced nuclear Nrf2 expression (Figure 6D).

In conclusion, these findings suggest that UA-NSps effectively prevented ANIT-induced liver damage in mice, primarily by suppressing the oxidative stress and activating the Nrf2/HO-1 signaling pathway.

### 3.9 UA-NSps alleviate ANIT-induced cholestasis by upregulating the expression of bile acid metabolism enzyme UGT2B1, transporter BSEP and MRP2

**FIGURE 7:**
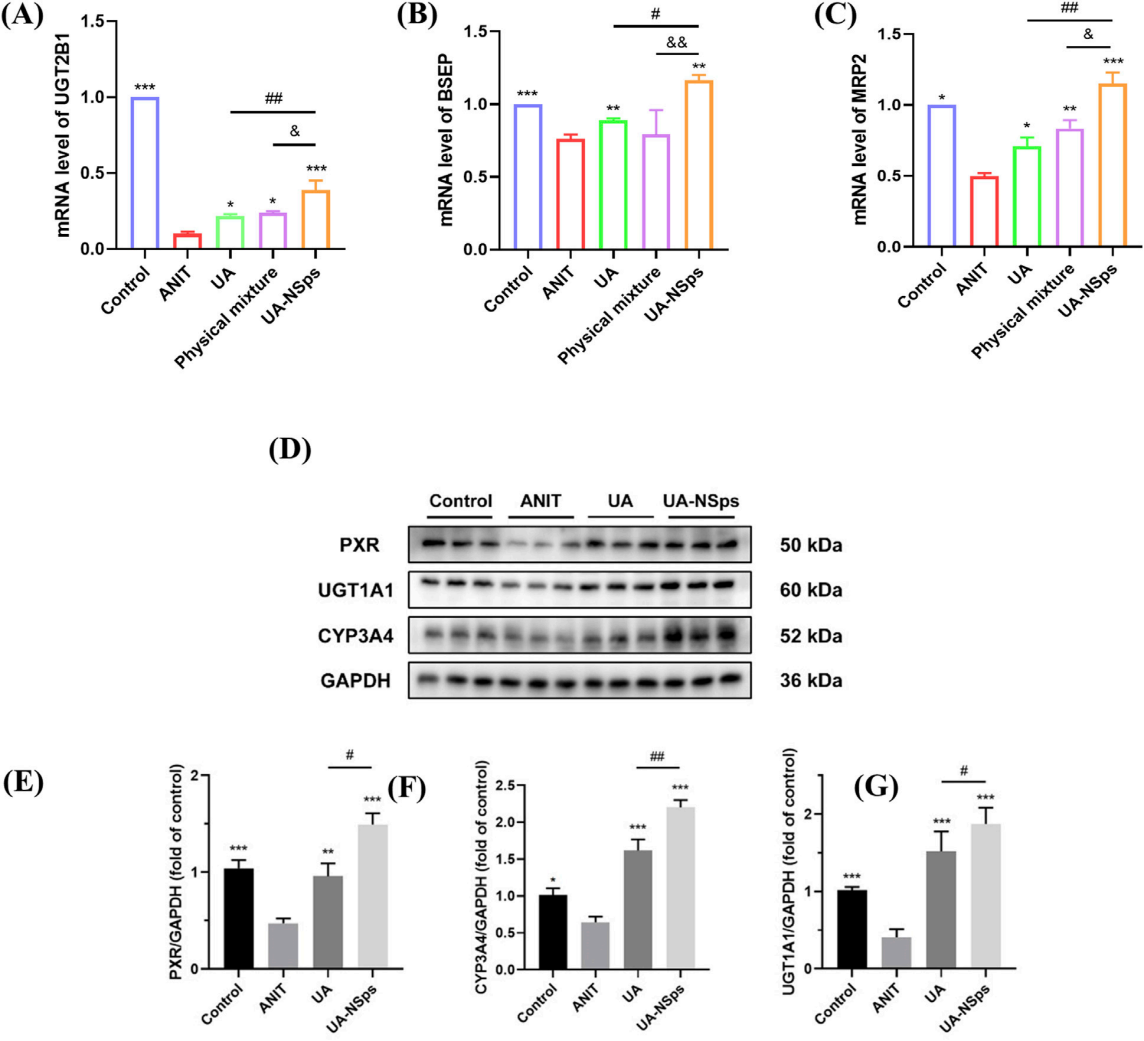
UA-NSps alleviate ANIT-induced cholestasis by regulating bile acid metabolism enzymes and the PXR/CYP3A4/UGT1A1signaling pathway. **(A–C)** The relative mRNA expression levels of UGT2B1, BSEP and MRP2. **(D)** Immunoblot assays of PXR, CYP3A4, and UGT1A1. Relative densities of **(E–G)** PXR, CYP3A4 and UGT1A1. Data are presented as the mean ± SEM (n = 3). Significant differences compared with the ANIT group, *p < 0.05, **p < 0.01, ***p < 0.001; compared with the UA group, ^#^ *p* < 0.05, ^##^ *p* < 0.01.

UGT2B1 is an important UDP-glucuronosyltransferase that plays a key role in bile acid metabolism. After bile acids are metabolized by UGT2B1 in the liver, they are excreted via the bile ducts by the combined action of BSEP and MRP2 transporters (Kasteel et al., 2020). The RT-qPCR results, shown in Figures 7A–C, indicated that, compared with the normal group, the ANIT group exhibited a significant downregulation of UGT2B1, BSEP, and MRP2 mRNA expression levels. In comparison to the ANIT group, all treatment groups showed significant upregulation of UGT2B1 and MRP2 mRNA expression levels (*p <* 0.05). Among them, the UA-NSps group showed a significantly better therapeutic effect than both the UA group and the physical mixture group (*p* < 0.05).

### 3.10 UA-NSps alleviate ANIT-induced cholestasis via the PXR/CYP3A4/UGT1A1 pathway

UGT1A1 plays a crucial role in the timely elimination of toxic bile acids, thereby effectively alleviating cholestasis (Kasteel et al., 2020). Similarly, CYP3A4 is a key metabolic enzyme that converts hydrophobic bile acids into hydrophilic bile acids (Lan et al., 2023). The PXR acts as a vital transcriptional regulator that induces the expression of CYP3A4 and regulates UGT1A1 (Chen et al., 2014). As shown in Figures 7D–G, the levels of PXR, CYP3A4, and UGT1A1 were significantly reduced in the ANIT group compared to the control group (*p* < 0.05). Administration of UA slightly increased the expression of these proteins. However, treatment with UA-NSps significantly elevated the levels of PXR, CYP3A4, and UGT1A1 (*p* < 0.05). These findings highlight the superior regulatory effects of UA-NSps on bile acid metabolism-related proteins, suggesting its potential to ameliorate cholestasis more effectively than UA alone.

## 4 Discussion

Cholestasis is a liver dysfunction closely associated with oxidative stress and bile acid (BA) metabolic disorders (Gasmi and Kleiner, 2020; Smets et al., 2021). Currently, due to the lack of effective therapies for cholestasis, it is urgent to develop safe and effective therapeutic agents and strategies. In this study, we developed a novel UA-VES drug-drug nanocrystal that not only enhances the bioavailability of UA but also targets oxidative stress and bile acid metabolism for synergistic treatment of CLI. Our findings provide a new therapeutic agent and strategy to treat cholestasis disease.

Currently, UA nanocrystals are mainly prepared using synthetic stabilizers, such as hydroxypropyl methylcellulose (HPMC) (Pi et al., 2016), Poloxamer 188 (P188) (Jacob et al., 2020), and polyvinyl alcohol (PVA) (Wang et al., 2015), which do not have pharmacological effects. It has been reported that vitamin E succinate (VES), with antioxidant, anti-inflammatory, and anti-fibrotic properties (Kim et al., 2016; Lan et al., 2021), could effectively alleviate liver damage combined with other medicines (Pietu et al., 2012), and could also act as a stabilizer in nanocrystals (Liu et al., 2012). In this study, we used VES as a stabilizer to prepare UA-NSps drug-drug nanocrystals. Pharmacological results showed that the serum levels of ALT, AST, ALP, and γ-GT in the physical mixture group were lower than those in the UA group, but no significant difference was observed (*p* > 0.05). This suggests that VES could play a synergistic role, but due to its low content, no significant difference was detected. Further investigations indicated that, in terms of improving liver histopathology and serum markers, the UA-NSps group had better therapeutic efficacy than free UA or physical mixture. This improvement is likely attributed to the increased dissolution rate and bioavailability of UA after nanoformulation, which enhanced its therapeutic efficacy.

Studies have demonstrated that inflammation, lipid peroxidation, and oxidative stress induced by toxic bile acid retention are present in both ANIT-induced cholestatic animal models and cholestatic patients (Yang et al., 2019; Zhang et al., 2019). Our research found that ANIT exposure significantly increased the pro-inflammatory cytokines IL-6 and TNF-α, exacerbating liver injury. However, UA-NSps effectively reversed the expression of these pro-inflammatory factors. ANIT exposure resulted in a significant increase in hepatic MDA levels in mice, along with a marked suppression of antioxidant enzymes SOD, GSH, and CAT activities. In contrast, UA-NSps treatment significantly alleviated the inhibitory effects of ANIT on antioxidant enzyme activity and reduced MDA levels. The antioxidant enzyme levels in the physical mixture group were slightly higher than in the UA group, but no significant difference was observed (*p* > 0.05), which may be attributed to the anti-inflammatory and antioxidant properties of VES, contributing to a synergistic effect. Importantly, UA-NSps exhibited a significantly stronger effect than either the UA group or the physical mixture group in restoring pro-inflammatory cytokine and antioxidant enzyme levels.

Numerous studies have shown that Nrf2 is a transcription factor and a key regulator of hepatic detoxifying enzymes, antioxidant stress genes, and many transporters, including members of the MRP family, serving as a cellular stress sensor (Shu et al., 2019; Robledinos-Antón et al., 2019; Zhang et al., 2020). Recent research indicates that UA can regulate bile acid metabolism through the Nrf2/UGT2B7/BSEP/MRP2 pathway to treat CLI (Wang et al., 2024). Our study also confirmed that UA-NSps enhanced Nrf2 nuclear translocation, upregulated its protein expression, increased the protein expression of the downstream antioxidant enzyme HO-1, and thereby provided protection against oxidative stress. Additionally, UA-NSps increased the expression of phase II detoxifying enzymes UGT2B7 and UGT1A1, as well as the transcriptional regulation of bile acid transporters BSEP and MRP2. Notably, UA-NSps treatment demonstrated stronger regulatory effects on these proteins than the UA group. This is likely due to the antioxidant properties of VES, which synergistically enhanced the ability of UA to activate Nrf2 and its downstream detoxifying enzymes. The results from this study indicate that UA-NSps significantly improve the therapeutic efficacy of UA and its antioxidant stress effect, driven both by the synergistic effects of VES and the enhanced dissolution rate and bioavailability of UA after nanoformulation.

Recent evidence suggests that the primary feature of cholestatic liver diseases is the accumulation of toxic components, such as hydrophobic bile acids, within hepatocytes (Gasmi and Kleiner, 2020). As an endogenous ligand of PXR, bile acids activate PXR, which in turn regulates the expression of downstream detoxifying enzymes, such as CYP3A4 and UGT1A1, as well as bile acid transporters including MRP and MDR1, to modulate bile acid clearance (Lan et al., 2023). CYP3A4 not only catalyzes the metabolic clearance of various exogenous substances, such as drugs, but also participates in the oxidative metabolism of many endogenous compounds, including hormones and bile acids (Chen et al., 2014).Studies have shown that ursolic acid (UA) can dose-dependently activate the mRNA and protein expression of PXR in HepG2 cells, although no *in vivo* studies have been reported (Kadasah and Radwan, 2023). Our research found that in the ANIT-induced cholestatic mouse model UA-NSps significantly upregulated the expression of PXR, CYP3A4, and UGT1A1 proteins in the liver, promoting the detoxification of toxic bile acids and maintaining the balance of bile acid metabolism in the body.

Other studies have reported that UA contains hydroxyl groups, which can be sulfated by CYP3A4 in intestinal epithelial cells, thereby reducing its bioactivity. Intestinal CYP3A4 affects the oral bioavailability of UA (Jinhua et al., 2020). However, we did not investigate the effects of UA-NSps on the transcription and protein expression of CYP3A4 in the mouse intestine, which could be explored in future studies.The rate-limiting enzyme cholesterol 7α-hydroxylase (CYP7A1), UGT1A1, BSEP, and MRP2, along with the sodium-taurocholate cotransporting polypeptide (NTCP), collectively mediate the synthesis, metabolism, transport, and reabsorption of bile acids in hepatocytes (Alamoudi et al., 2021). Our study primarily focused on the mechanisms by which UA-NSps influence bile acid metabolism and transport, but we did not investigate their effects on bile acid synthesis, which represents a limitation of the study and an area for future research.

## 5 Conclusion

**FIGURE 8:**
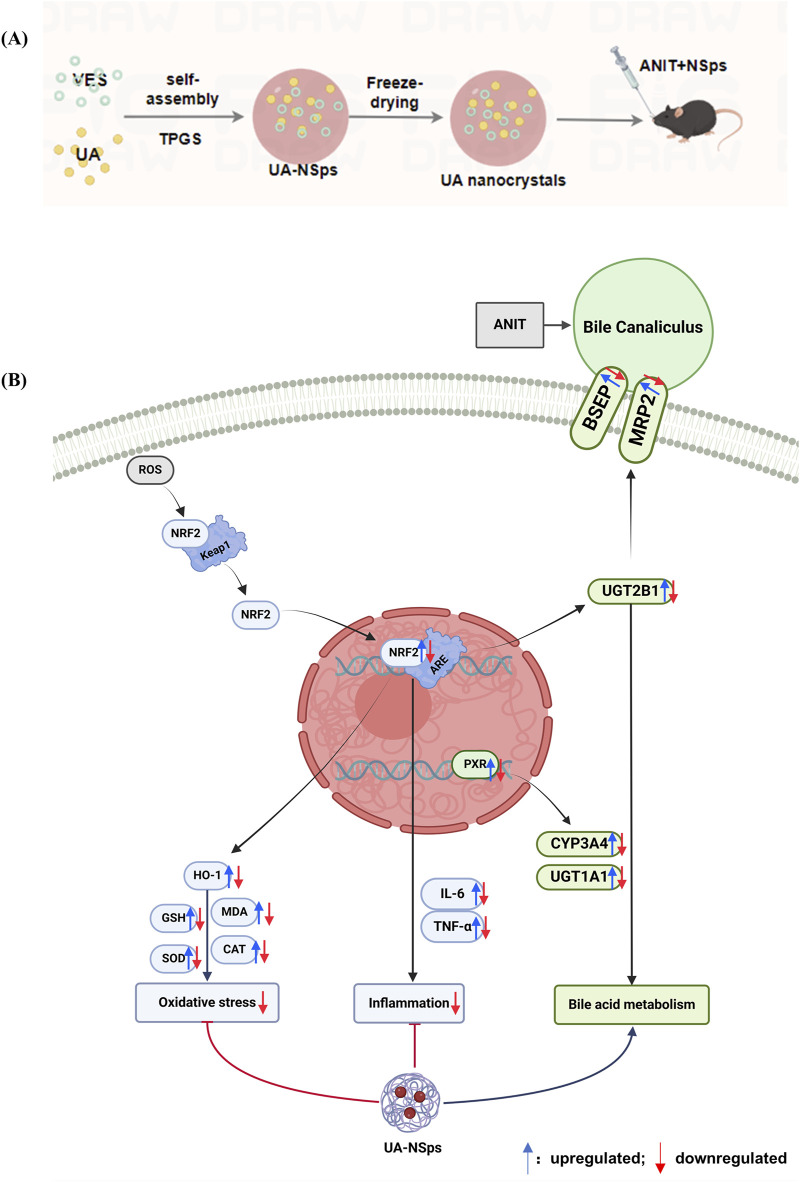
Schematic representation of the preparation of UA-NSps **(A)** and its therapeutic mechanism in ANIT-induced cholestatic liver injury **(B)**.

The preparation of UA-NPs and their mechanism in ameliorating cholestatic liver injury are summarized in Figure 8. In this research, we successfully developed a UA and VES drug-drug nanocrystals (UA-NSps) via the anti-solvent precipitation and freeze-drying method. UA-NSps exhibited high drug—loading capacity, spherical morphology, and improved dissolution and oral bioavailability. In the ANIT model, UA-NSps effectively restored liver function, as demonstrated by histopathological and biochemical improvements. Mechanistically, UA-NSps enhanced Nrf2 nuclear translocation, upregulated Nrf2 and its downstream effector HO-1, reduced pro-inflammatory cytokines, and ameliorated mitochondrial damage. Moreover, UA-NSps alleviated bile acid metabolism and transport disorder-induced hepatic cholestasis by upregulating the transcriptional activity of bile acid metabolic enzyme UGT2B1 and transporters BSEP and MRP2, as well as the nuclear receptor and metabolic enzymes PXR/CYP3A4/UGT1A1. Our study presents a novel drug-drug nanosuspension preparation strategy that enhances oral bioavailability while synergistically improving the treatment of cholestatic liver injury (CLI).

## Data Availability

The datasets presented in this study can be found in online repositories. The names of the repository/repositories and accession number(s) can be found in the article/Supplementary Material.
