# Supplementary material for: Ursolic acid drug-drug nanocrystals ameliorate cholestatic liver injury via inhibiting oxidative stress and regulating bile acid metabolism
Source: Front Pharmacol. 2025 May 30;16:1586141. doi: 10.3389/fphar.2025.1586141 (PMC12162325; doi:10.3389/fphar.2025.1586141)
Supplement: Supplementary file 1 [file DataSheet1.pdf]

Supplementary information

Ursolic Acid drug-drug nanocrystals ameliorate cholestatic liver injury via inhibiting oxidative stress and regulating bile acid metabolism

Manhang Hu[a,1], Xiaolu Hua[a,b,1],Wei Xiong[c], Enhui Zheng[a], XingYueYang[a] , Yu Lu[a], Bing He[d], XiaolinZhong[e], ZongZhe Jiang[a], Qingbi Zhang[a,b*], YanLiu[a*]

a. Basic Medicine Research Innovation Center for cardiometabolic diseases, Ministry of Education, Luzhou Municipal Key Laboratory of Thrombosis and Vascular Biology, Metabolic Vascular Diseases Key Laboratory of Sichuan Province, School of Pharmacy, Southwest Medical University, Luzhou, 646000, PR China.
b. Environmental health effects and risk assessment Key Laboratory of Luzhou, School of Public Health, Southwest Medical University, Luzhou, 646000, PR China.
c. Department of Science and Technology, Southwest Medical University, Luzhou, 646000, PR China.
d. The Public Platform of Advanced Detecting Instruments, Public Center of Experimental Technology, Southwest Medical University, Luzhou 646000, China
e. Department of Gastroenterology,The Affiliated Hospital of Southwest Medical University, Luzhou, 646000, PR China.

*Corresponding authors. E-mail addresses: liuyanlucky@swmu.edu.cn (Yan Liu) qingbizhang@swmu.edu.cn (Qingbi Zhang)

1. These authors contributed to the work equally and should be regarded as co-first authors.

Table 1 Process optimization of UA-NSps (n=3)

a. Solvent Optimization of UA

Solvent	Size(nm)	PDI	Zeta(mV)
ETOH	237.9±3.329	0.287±0.026	-21±0.551
DMSO	169.4±0.839	0.137±0.026	-8.07±1.94
MEOH	202±1.87	0.22±0.025	-20.6±1.87

b. The effect of the ratio of VES and UA on UA-NSps (n=3)

Ratio(VES/UA)	Size(nm)	PDI	Zeta(mV)
0.2	269.1±5.06	0.421±0.036	-20.2±0.173
0.4	199.4±1.662	0.242±0.017	-10.3±1.4
0.6	204.5±1.365	0.23±0.016	-19.9±1.5
1	303.1±2.954	0.443±0.014	-19.6±7.17

c. Influence of different amount of TPGS on UA-NSps (n=3)

VES(mg)	UA(mg)	TPGS (mg)	Size(nm)	PDI	Zeta(mV)	UA concentration (mg/ml)
1.8	3	0.2	164.9±2.458	0.144±0.008	28.2±1.2	0.131
		0.5	183.4±2.73	0.181±0.011	-30±1.62	0.121
		1	183±1.75	0.161±0.009	-22.5±0.55	0.182
		1.5	181±0.3464	0.194±0.006	-21.9±3.14	0.136
		2	178.7±0.651	0.142±0.008	0.769±1.38	0.158

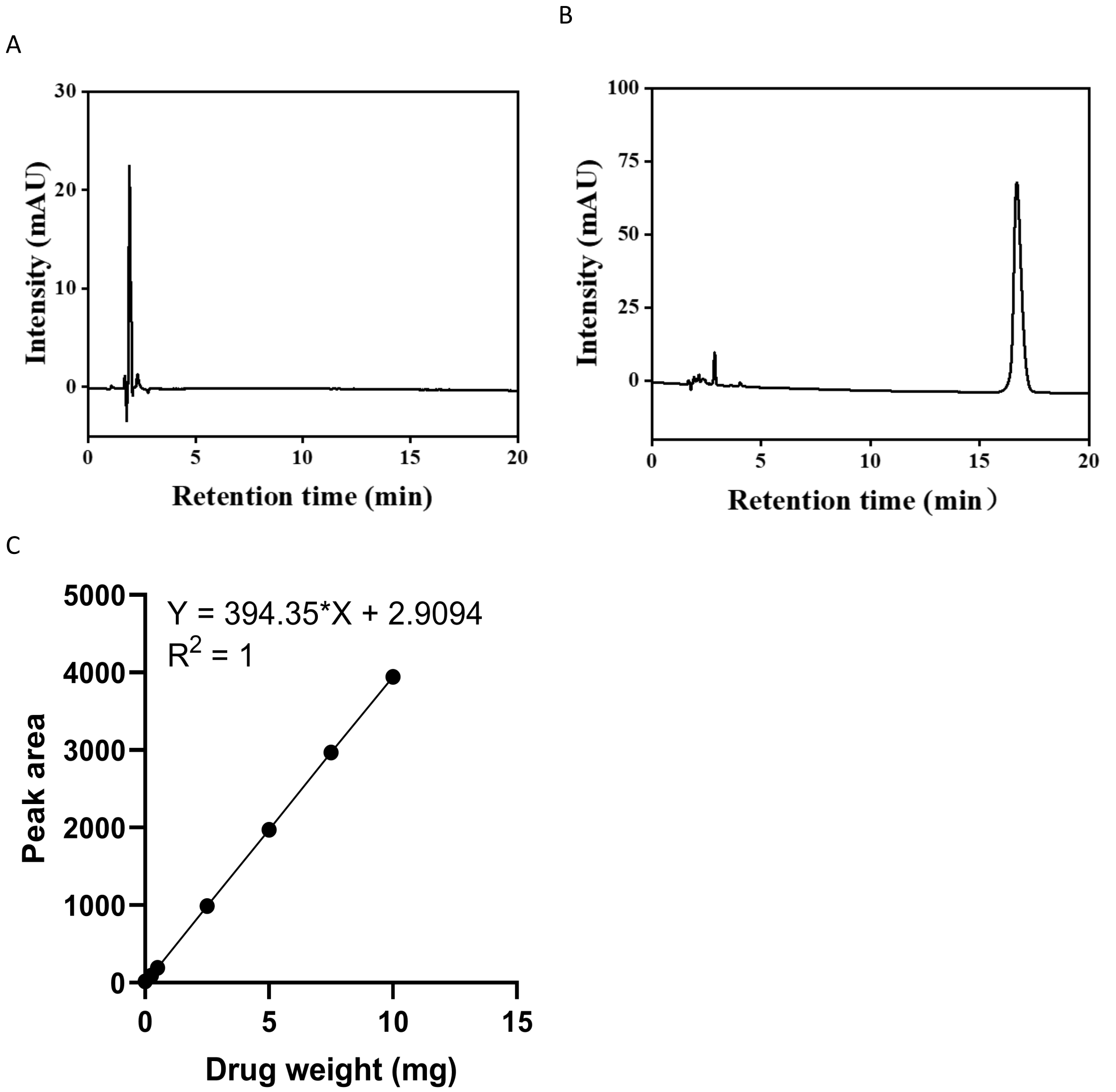

Figure S1 **A** The HPLC chromatograms of blank solvent. **B** The HPLC chromatograms of Ursolic acid reference solution. **C** The standard curve of Ursolic acid

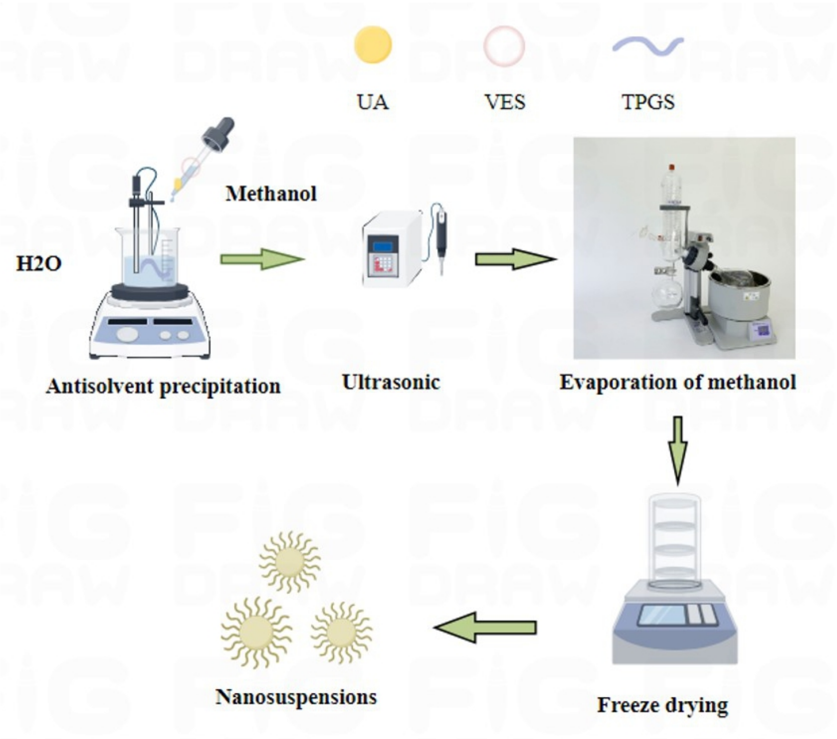

Fig. S2 The preparation process of UA-NSps

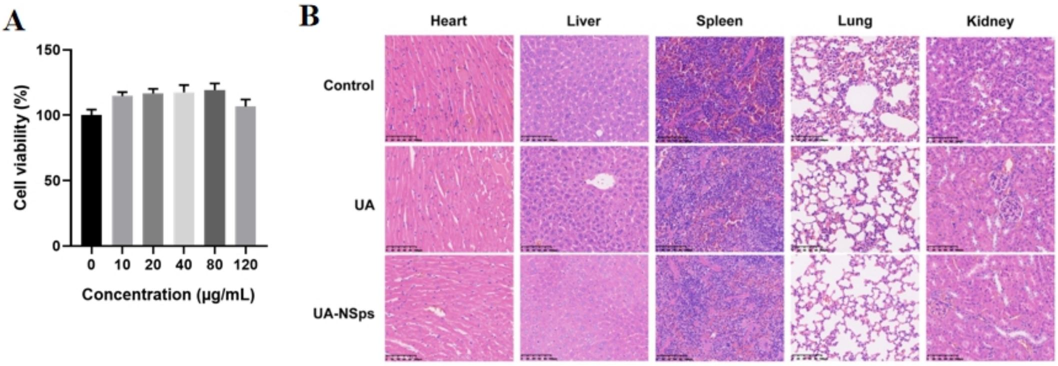

Fig. S2 Drug safety evaluation. A Effect of different concentrations of UA-NSps on the viability of LO2 cells . Data are expressed as the mean ± SD (n=3). B HE staining . Scale bar were 100 μm

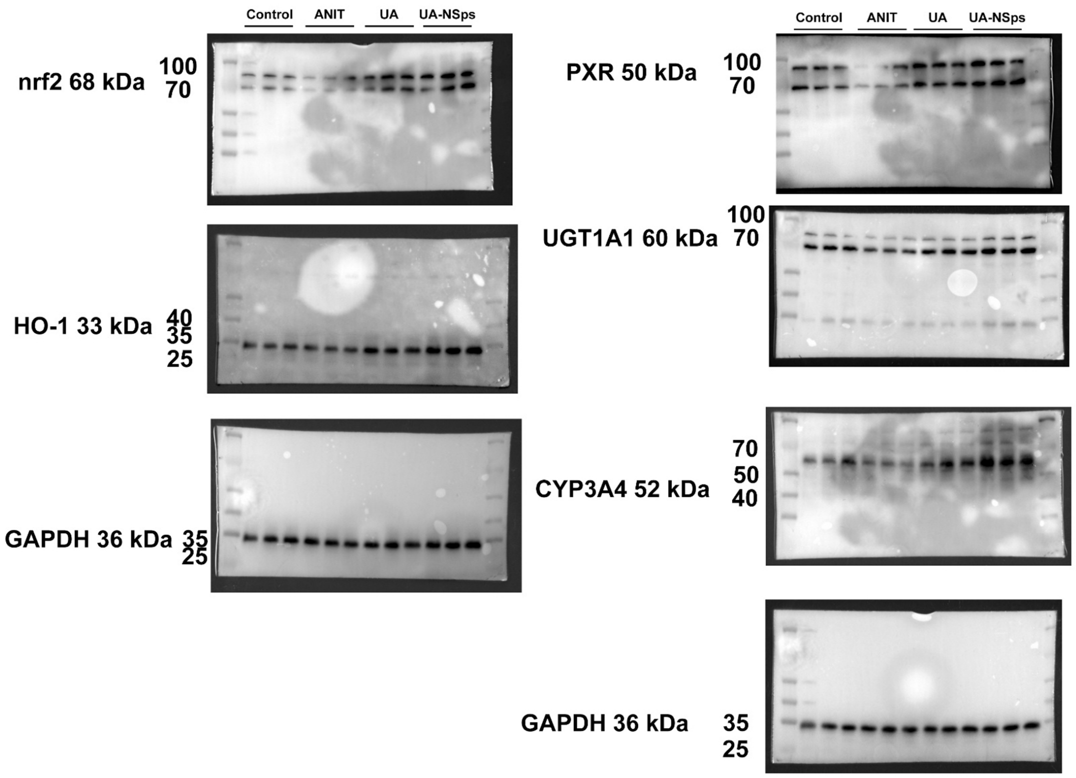

Fig. S4 The whole un-cropped images of the original western blots

Tab.2 Primer sequence

Primer	Forward	Reverse
UGT2B1	TTGATGTCGTTCTAGCAGATGC	TCAAAAAGCAACACCTGCAAC
MRP2	GCACTGTAGGCTCTGGGAAG	CATTTCCAAGTCTGGGAGGA
BSEP	TCTGACTCAGTGATTCTTCGCA	CCCATAAACATCAGCCAGTTGT
GAPDH	GGTTGTCTCCTGCGACTTCA	TGGTCCAGGGTTTCTTACTCC
